# Supplementary material for: Unsupervised-learning-based method for chest MRI–CT transformation using structure constrained unsupervised generative attention networks
Source: Sci Rep. 2022 Jun 30;12:11090. doi: 10.1038/s41598-022-14677-x (PMC9247083; doi:10.1038/s41598-022-14677-x)
Supplement: Supplementary file 2 — Supplementary Legends. [file 41598_2022_14677_MOESM2_ESM.docx]

**Supplementary material**

An animation of 3D VR images composited from the synthesised CT is submitted as "Supplementary Material" along with this manuscript.

The pair plots of the visual evaluation scores by radiologists for CycleGAN. CycleGAN + MIND, U-GAT-IT, and U-GAT-IT + MIND.

[Sup. Figure 1 near here]

[Sup. Figure 2 near here]

[Sup. Figure 3 near here]

[Sup. Figure 4 near here]

**Supplementary material legends**

**Animation 1** An animation of 3D VR images composited from the synthesised CT.

**Sup. Figure 1** Pair-plot of visual evaluation scores (Dr. A) (dark circles indicate high frequencies).

**Sup. Figure 2** Pair-plot of visual evaluation scores (Dr. B) (dark circles indicate high frequencies).

**Sup. Figure 3** Pair-plot of visual evaluation scores (Dr. C) (dark circles indicate high frequencies).

**Sup. Figure 4** Pair-plot of visual evaluation scores (Dr. D) (dark circles indicate high frequencies).
