# Supplementary figures and images for: Unsupervised-learning-based method for chest MRI–CT transformation using structure constrained unsupervised generative attention networks
Source: Sci Rep. 2022 Jun 30;12:11090. doi: 10.1038/s41598-022-14677-x (PMC9247083; doi:10.1038/s41598-022-14677-x)

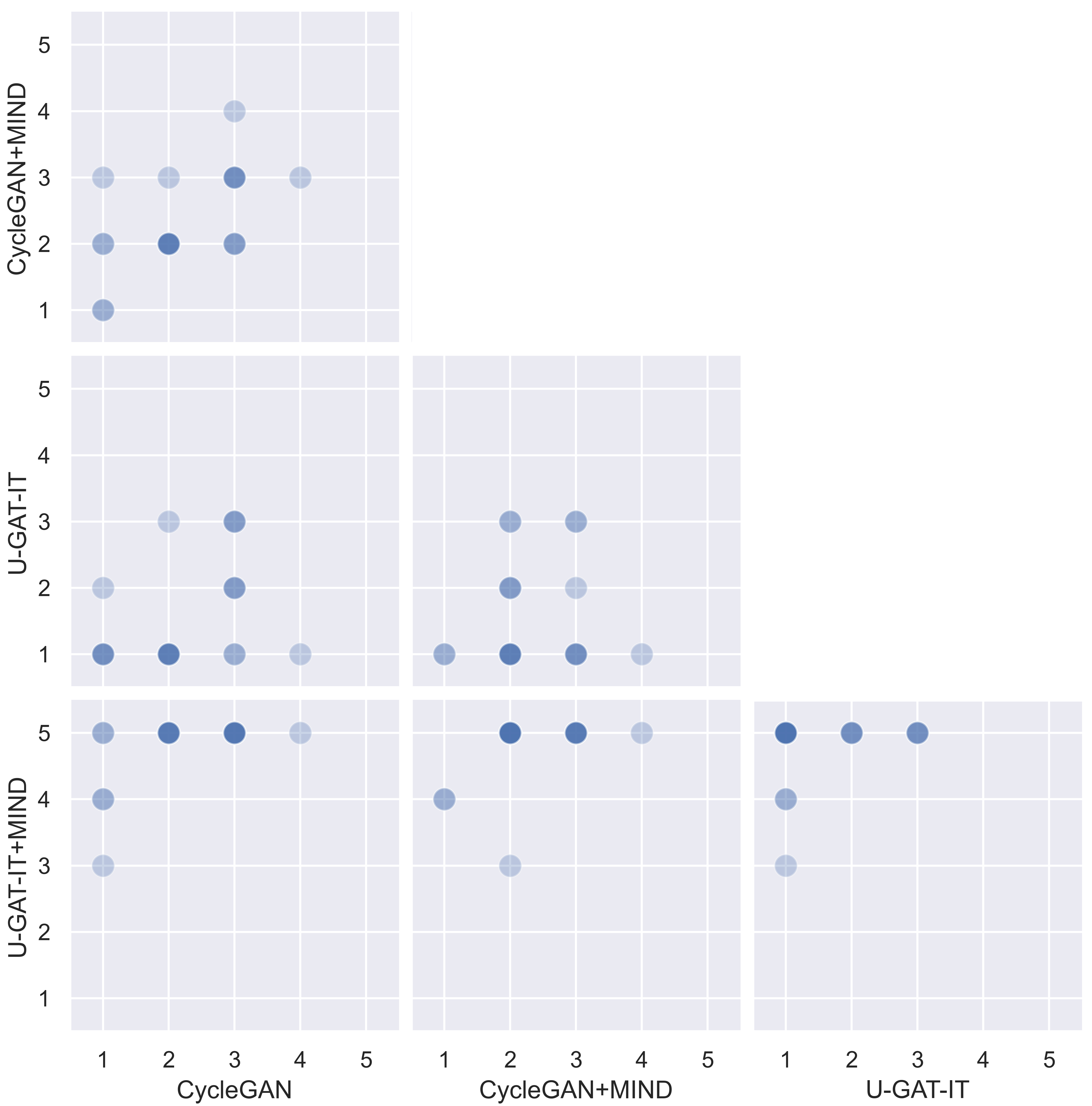

Supplement: Supplementary file 3 — Supplementary Figure 1. [file 41598_2022_14677_MOESM3_ESM.png]

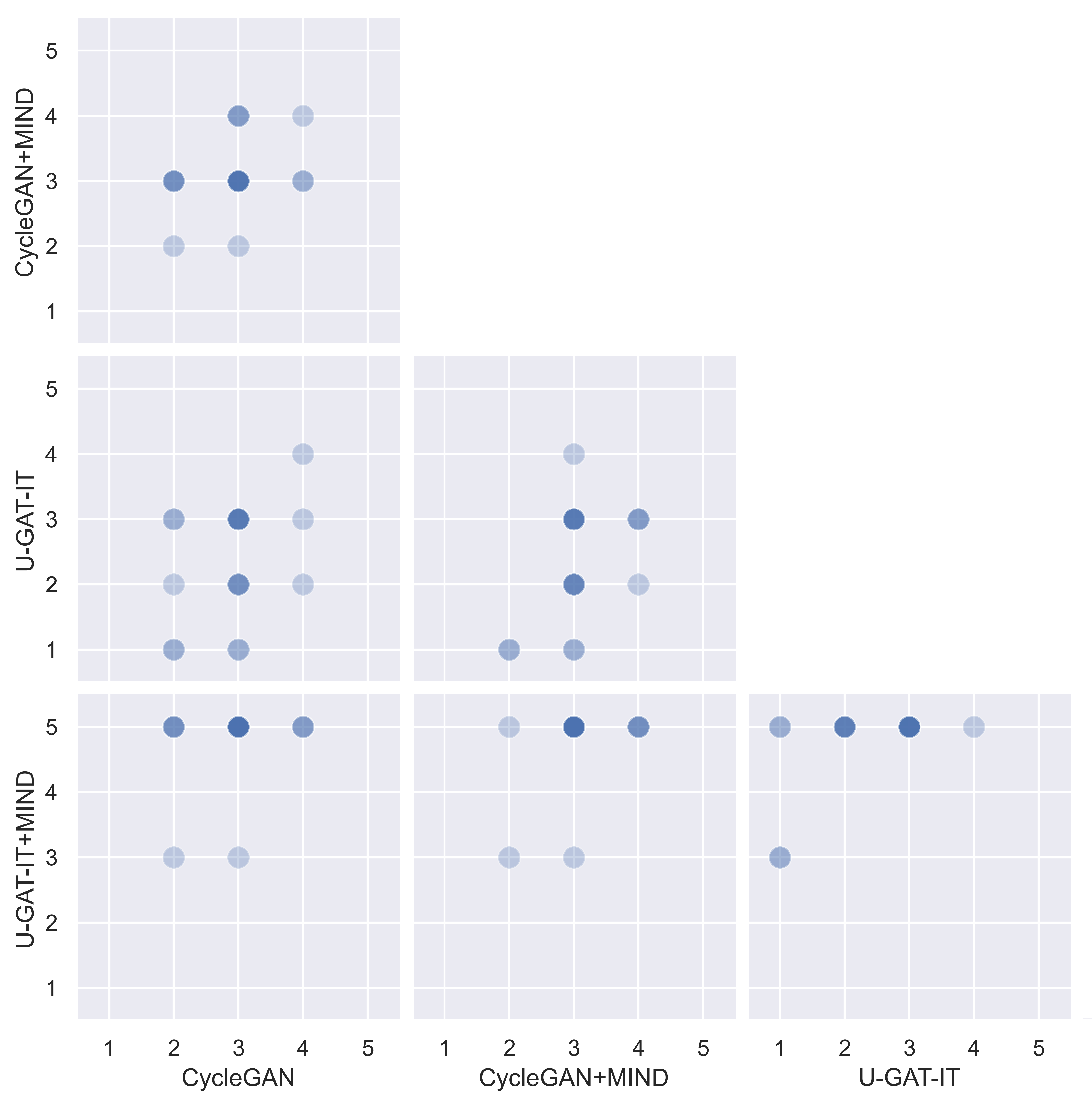

Supplement: Supplementary file 4 — Supplementary Figure 2. [file 41598_2022_14677_MOESM4_ESM.png]

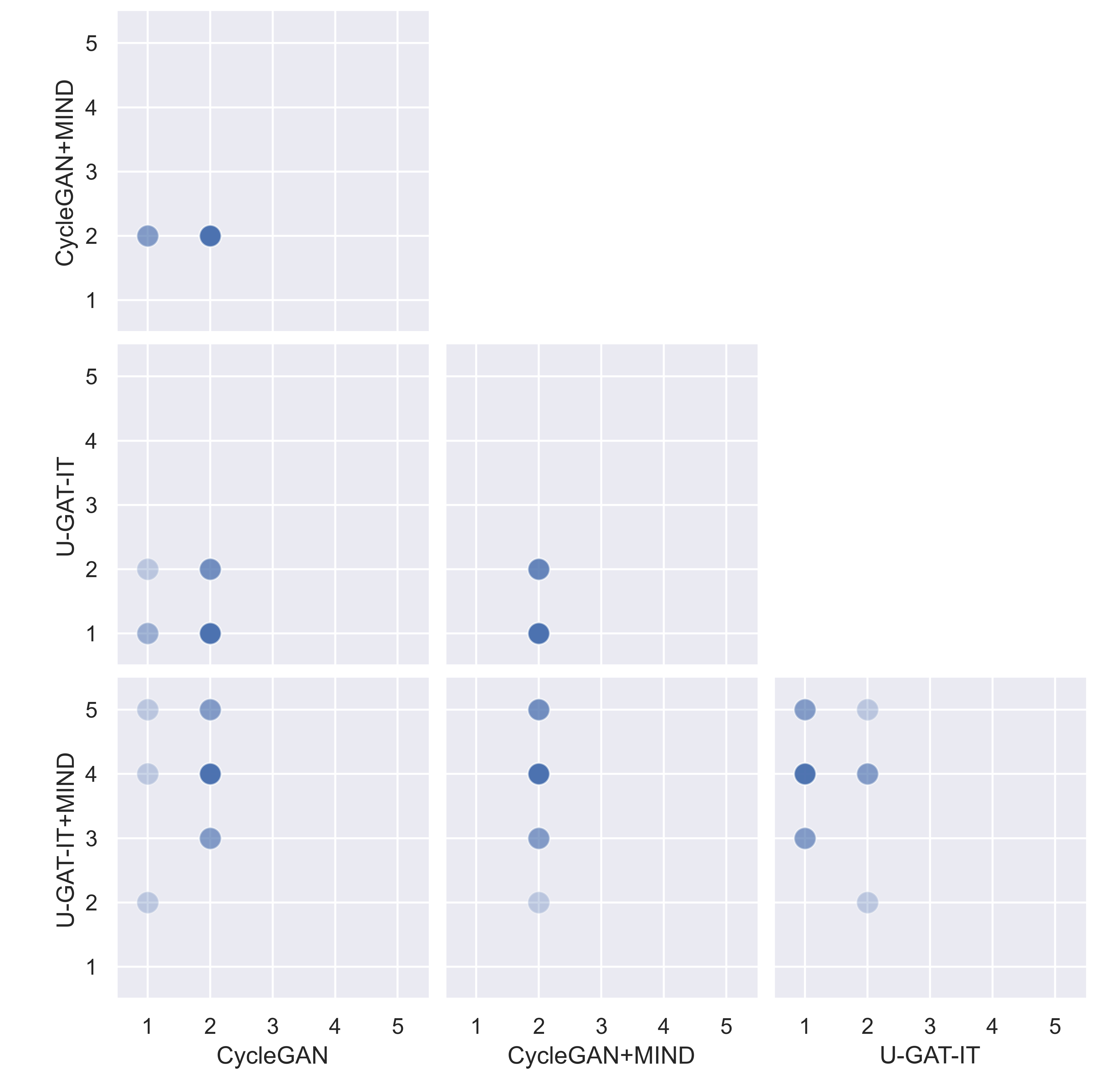

Supplement: Supplementary file 5 — Supplementary Figure 3. [file 41598_2022_14677_MOESM5_ESM.png]

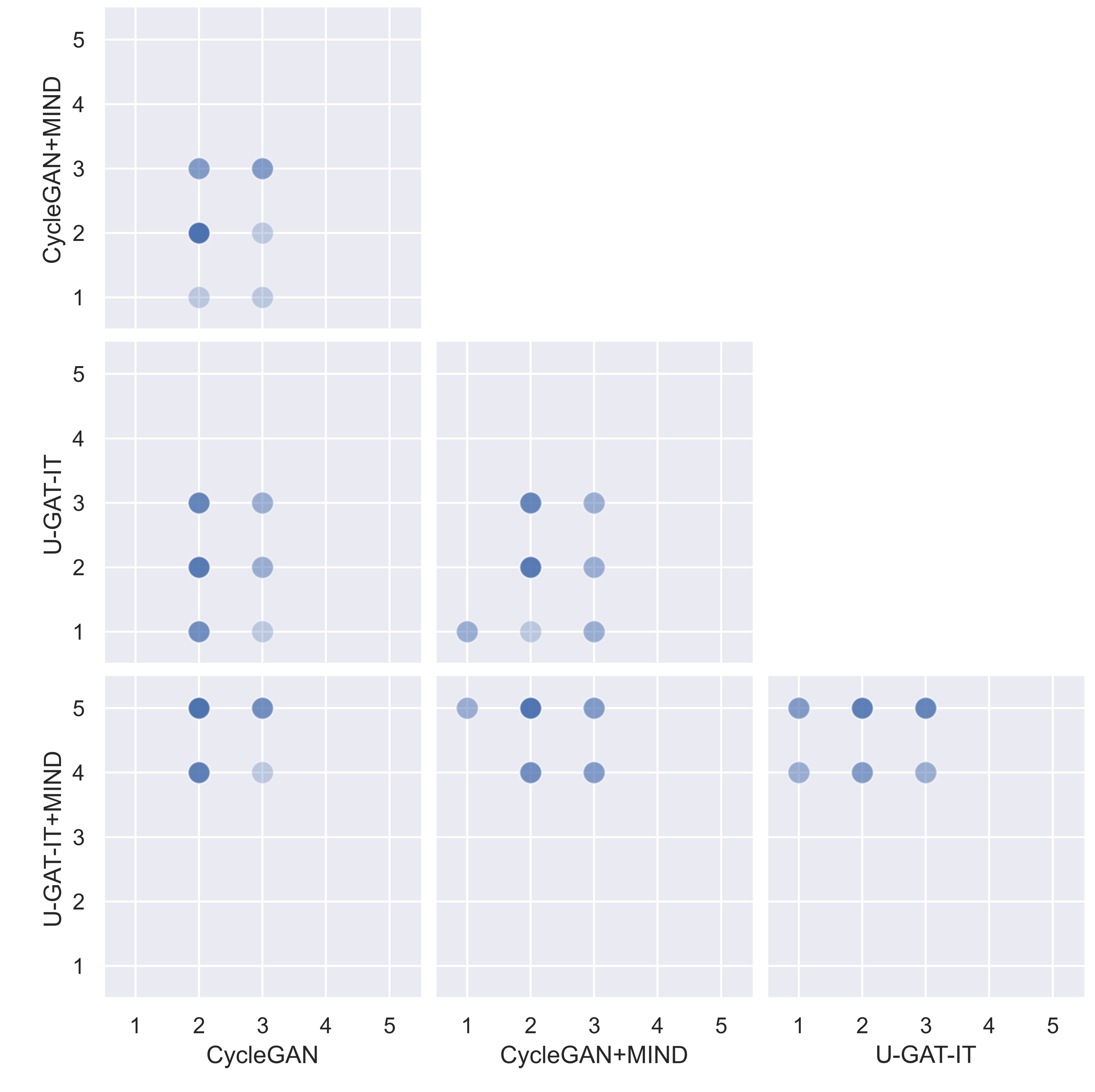

Supplement: Supplementary file 6 — Supplementary Figure 4. [file 41598_2022_14677_MOESM6_ESM.png]
